# Sensing data and methodology from the Adaptive DBS Algorithm for Personalized Therapy in Parkinson’s Disease (ADAPT-PD) clinical trial

**DOI:** 10.1038/s41531-024-00772-5

**Published:** 2024-09-17

**Authors:** Scott Stanslaski, Rebekah L. S. Summers, Lisa Tonder, Ye Tan, Michelle Case, Robert S. Raike, Nathan Morelli, Todd M. Herrington, Martijn Beudel, Jill L. Ostrem, Simon Little, Leonardo Almeida, Adolfo Ramirez-Zamora, Alfonso Fasano, Travis Hassell, Kyle T. Mitchell, Elena Moro, Michal Gostkowski, Nagaraja Sarangmat, Helen Bronte-Stewart, On behalf of the ADAPT-PD Investigators

**Affiliations:** 1 Medtronic Neuromodulation, Medtronic, Minneapolis, Minnesota USA; 2 Massachusetts General Hospital, Harvard Medical School, Boston, USA; 3 Department of Neurology, Amsterdam University Medical Centers, Amsterdam, Netherlands; 4 Department of Neurology, University of California San Francisco, San Francisco, USA; 5 Department of Neurology, University of Minnesota, Minneapolis, USA; 6 Department of Neurology, Shands at University of Florida, University of Florida, Gainesville, USA; 7 Edmond J. Safra Program in Parkinson’s Disease, Morton and Gloria Shulman Movement Disorders Clinic, Toronto Western Hospital, UHN, University of Toronto, Toronto, ON Canada; 8 Krembil Brain Institute, University of Toronto, Toronto, ON Canada; 9 Department of Neurology, Vanderbilt University Medical Center, Nashville, USA; 10 Duke University Movement Disorders Center, Duke University, Durham, USA; 11 Grenoble Alpes University, Division of Neurology, Grenoble Institute of Neuroscience, CHU of Grenoble, Grenoble, France; 12 Center for Neurological Restoration, Cleveland Clinic Foundation, Cleveland, USA; 13 Oxford University Hospitals NHS Foundation Trust, Oxford, UK; 14 Department of Neurology and Neurological Sciences, Stanford University, Stanford, USA

**Keywords:** Parkinson's disease, Parkinson's disease

## Abstract

Adaptive deep brain stimulation (aDBS) is an emerging advancement in DBS technology; however, local field potential (LFP) signal rate detection sufficient for aDBS algorithms and the methods to set-up aDBS have yet to be defined. Here we summarize sensing data and aDBS programming steps associated with the ongoing Adaptive DBS Algorithm for Personalized Therapy in Parkinson’s Disease (ADAPT-PD) pivotal trial (NCT04547712). Sixty-eight patients were enrolled with either subthalamic nucleus or globus pallidus internus DBS leads connected to a Medtronic Percept^TM^ PC neurostimulator. During the enrollment and screening procedures, a LFP (8–30 Hz, ≥1.2 µVp) control signal was identified by clinicians in 84.8% of patients on medication (65% bilateral signal), and in 92% of patients off medication (78% bilateral signal). The ADAPT-PD trial sensing data indicate a high LFP signal presence in both on and off medication states of these patients, with bilateral signal in the majority, regardless of PD phenotype.

## Introduction

Deep brain stimulation (DBS) therapy for the treatment of Parkinson’s disease (PD) is considered an evidence-based standard of care therapy^1–3^ that treats the cardinal motor symptoms of PD and reduces medication induced motor complications^4,5^. However, there is an unmet need to improve clinical efficacy of DBS therapy as not all people with PD actualize the full therapeutic benefits^6–8^. Poor or variable patient responses to DBS therapy may serve as an impediment to wider adoption and patient access to DBS therapy. One reason that current DBS therapy may result in limited effectiveness for some patients is that each patient’s symptoms are dynamic and unique, and the options for the managing clinician to personalize DBS therapy are limited due to inherent restrictions of continuous DBS which does not respond to changing clinical states.

Technology advances have provided opportunities to personalize DBS therapy with directional leads delivering stimulation *where* it is needed and closed-loop or adaptive DBS (aDBS) algorithms delivering stimulation *when* it is needed. Delivering the right amplitude or “dose” of stimulation *when* it is needed, in an adaptive manner, may provide more stable symptom control for patients. This is especially needed across the continuum of care as motor complications and fluctuations increase throughout the disease process^9^. Hence, the clinical goal of aDBS is to provide stable symptom control by delivering stimulation in consort with combined medication therapies and automating stimulation amplitude adjustments based on changes in the individual patient’s brain state. The most promising control signal for aDBS currently is local field potential (LFP) beta (±13–30 Hz) band oscillatory power in the globus pallidus internus (GPi) or subthalamic nucleus (STN)^10^ due to its relationship with fluctuating parkinsonian symptoms^11–17^, responsiveness to medication and DBS therapy^18–20^ and presence in roughly 95% of patients in an off-medication state^10,21^. By delivering stimulation adaptively, based on the patient’s relative beta band power, aDBS is being designed to automatically adjust stimulation during “on” and “off” states (reflected by relatively low and high levels of beta power, respectively) in order to minimize medication need, improve wearing-off motor symptoms and reduce periods of dyskinesia from excessive therapy most importantly in STN-DBS (i.e., times of peak medication + DBS therapy).

The building consensus is that aDBS can be feasibly and safely implemented to deliver similar or more efficacious therapy as continuous DBS (cDBS) to manage cardinal PD motor symptoms, reduce side effects, while preserving neurostimulator battery longevity^22–25^. Overall, results from small-sample feasibility studies of aDBS using LFP alpha (8–13 Hz) and beta control signals suggest that most PD motor symptoms can be responsive to aDBS including bradykinesia^26–28^, rigidity^29^, tremor^27,30^, dyskinesia^28,29,31,32^ and freezing of gait^33^. Moreover, acute aDBS in people with PD has also been reported to deliver less stimulation energy during times of peak levodopa concentration compared to end of dose^34^ and provide more desirable effects on speech intelligibility^35^ and troublesome dyskinesia^32^. Short-term studies of aDBS in people with PD suggest an estimated ~48–74% reduction in stimulation energy required to achieve effective therapy compared to cDBS^26,27,29,30,32,36^; yet, there is still limited understanding of the potential energy savings in a community setting with chronic aDBS. In the semi-chronic studies to date, aDBS has also been well tolerated by freely-moving patients in and out of the clinical setting^29,31,37^. Despite these promising early results, there remains a lack of evidence regarding real-world and chronic use of aDBS in larger clinical cohorts.

Technological advancements and evidence from the acute and semi-chronic clinical studies of aDBS in PD over the past ~10 years have garnered sufficient confidence to feasibly implement chronic, at-home aDBS in a moderately-sized, multicenter clinical trial. Consequently, the ADAPT-PD clinical trial was designed to demonstrate the safety and effectiveness of chronic dual and single threshold aDBS modes (see “Methods” for a description of aDBS modes) in the community setting, and in all PD patient subtypes indicated for DBS. Whereas the primary cohort received omni-directional stimulation, a second cohort received directional stimulation which will provide the very first report of combined aDBS and directional stimulation in patients. In addition, the study data will generate a wealth of clinical and physiological data for assessing the different potential clinical advantages of aDBS in the domains of neurostimulation side effects, energy savings, PD motor symptom control, sleep quality, speech intelligibility, and quality of life. The results of the ADAPT-PD pivotal trial are also anticipated to support regulatory submissions for aDBS, which could be a significant advancement towards truly personalized treatment for people with PD treated with DBS therapy. Furthermore, this study is a critical step toward advancing closed-loop neuromodulation as a clinical approach for treating patients with other neurological and psychiatric disorders. The purpose of the present work is to disseminate the methods by which aDBS was programmed by clinicians during the ADAPT-PD trial and provide sensing data supporting the feasibility of recording LFP signals in a diverse cohort of people with PD.

## Results

The ADAPT-PD trial included 10 centers in the United States, Europe, and Canada enrolling participants since December 2020. Sixty-eight people with PD and bilateral DBS (51 STN and 17 GPi) were enrolled in the ADAPT-PD Primary Cohort (Table 1). Data from the Directional Cohort is not included in the present report. A total of 15 participants exited the study during the Enrollment Phase due to screen failure (*n* = 11), withdrawal by the participant (*n* = 3), clinician decision (*n* = 1).

**Table 1:** Patient demographics and DBS characteristics

Variable	Mean (SD) or *N* (%)
Age (years) (*n* = 66)	62.2 (8.4)
Sex (*n* = 68)	20 (29.4%) Females
Disease duration (years) (*n* = 64)	13.5 (6.8)
MDS-UPDRS-III (OFF Stim/off Med) (*n* = 58)	45.7 (14.9)
Clinical Subtype	Akinetic Rigid: 38 (65.5%)
	Tremor Dominant: 13 (22.4%)
	Mixed: 7 (12.1%)
Lead Type by subject (*n* = 68)	Legacy: 54 (79.4%)
	SenSight^TM^: 14 (20.6%)
DBS Target by subject (*n* = 68)	Bilateral—STN: 51 (75%)
	Bilateral—GPi: 17 (25%)

Note: All SenSight electrodes programmed in ring mode for patients in the Primary Cohort. Demographics calculated from data at enrollment with some missing data for age, disease duration, and MDS-UPDRS.

### LFP detection rate and characteristics

**Fig. 1:**
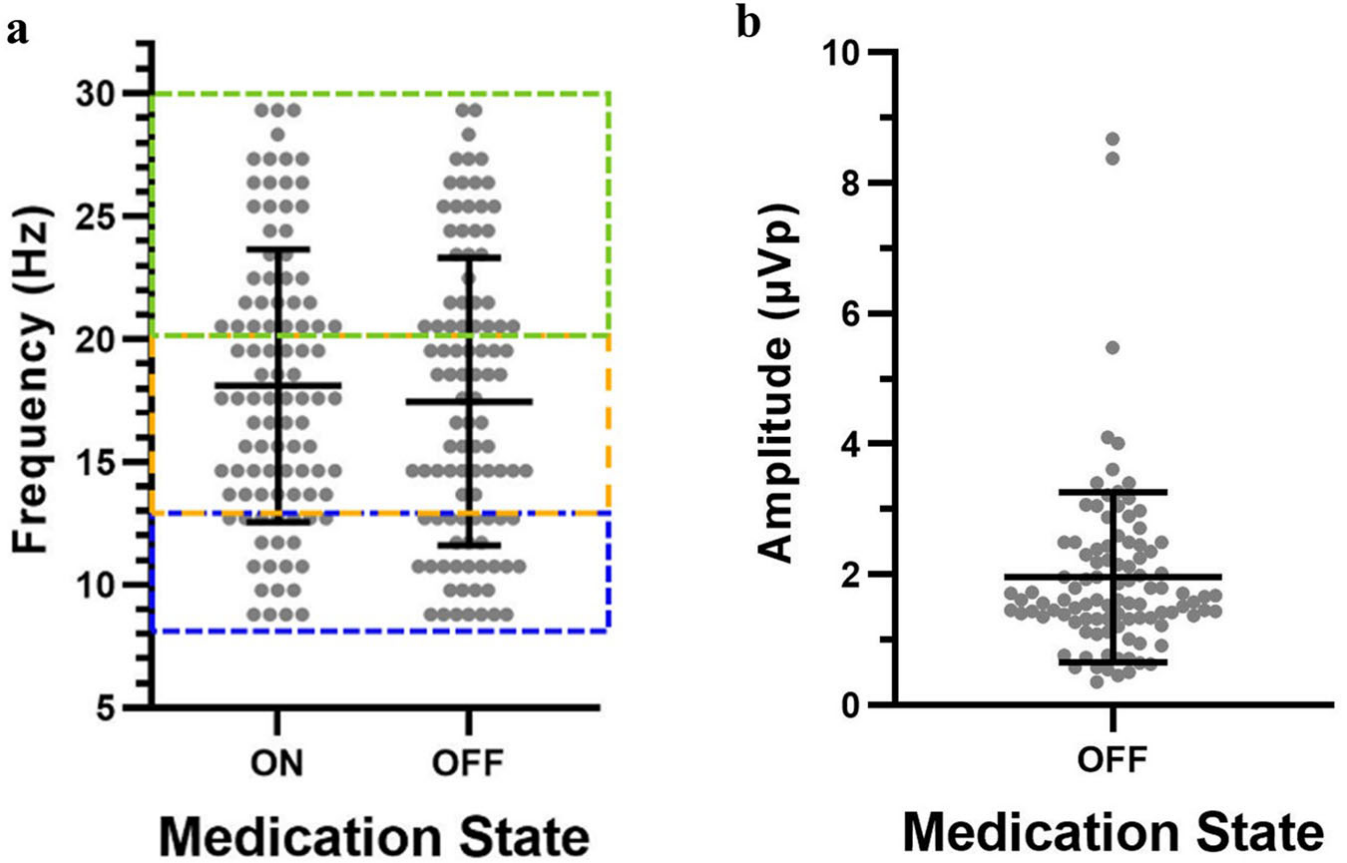
Clinician-identified local field potential (LFP) characteristics. **a** Peak LFP frequency on and off medication. **b** Peak LFP amplitude off medication. Signals combined between subthalamic nucleus and globus pallidus internus. Data included all hemispheres from subjects with unilateral or bilateral LFP signal meeting study eligibility criteria.

Table 2 summarizes the peak detection rate and frequency distribution by clinicians in the on and off medication state. Peak detection by clinicians occurred in 91.5% of participants off medication, and 84.8% on medication. Frequency of the peak LFP had the largest shift from medication on to off state in the alpha band (21.2% on medication, 31% off medication (Table 2 and Fig. 1a). Figure 1a illustrates the range of frequencies identified by clinicians in the on and off medication state, with the average LFP peak in the low beta range (off Meds: 17.5 ± 5.9 Hz; on Meds: 18.1 ± 5.5 Hz). The average LFP peak amplitude was 1.96 ± 1.3 µVp off medication (Fig. 1a). Of the 11 (16%) patients that did not have an LFP peak meeting inclusion criteria, six (9%) had signal artifact, four (6%) had an LFP peak power that was either too low or outside of the 8–30 Hz window, and one (1%) did not have any LFP data collected as they exited the study for other reasons.

**Table 2:** Characteristics Clinician-Identified LFP peaks

	On medication	Off medication
Peak detection		
Participants (*N*)	66	59
Peak detected	56 (84.8%)	54 (91.5%)
Bilateral peaks	43 (65.2%)	46 (78.0%)
Unilateral peak	13 (19.7%)	8 (13.6%)
No peaks	10 (15.2%)	5 (8.5%)
*Frequency band of peaks*		
Hemispheres (*N*)	99	100
Alpha	21 (21.2%)	31 (31.0%)
Low-beta	41 (41.4%)	34 (34.0%)
High-beta	37 (37.4%)	35 (35.0%)

Data from the signal check event during Enrollment Visit (on medication visit) and LFP Screening (off medication). Signals combined between subthalamic nucleus and globus pallidus internus. The on medication Enrollment visit had 2 missing datasets (*N* = 66), and nine subjects exited the study prior to the off medication LFP Screening visit (*N* = 59).

### STN and GPi LFP detection

An LFP peak meeting inclusion criteria occurred in 84% (57/68) of participants, corresponding to 40 patients with STN DBS (78% of STN patients) and 17 patients with GPi DBS (100% of GPi patients). Including all hemispheres with a detectable peak, STN peaks were distributed among alpha (15.3% on medication, 19.7% off medication), low-beta (43.1% on medication, 43.7% off medication), and high-beta (41.7% on medication, 36.6% off medication) range. GPi peaks were distributed among alpha (37% on medication, 58.6% off medication), low-beta (37% on medication, 10.3% off medication), and high-beta (25.9% on medication, 31% off medication).

### Automated algorithm LFP detection

**Fig. 2:**
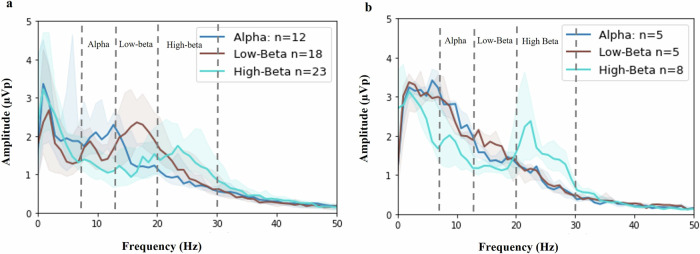
Automated algorithm-identified peaks in 71 nuclei and 45 patients on medication. **a** STN, (*N* = 53) and **b** GPi, (*N* = 18) peaks. The median and interquartile range power spectral density (PSD) are shown. PSDs are categorized by frequency: alpha (dark blue), low-beta (red) and high-beta (teal) bands plotted together.

On medication, LFP data were available for off-line analysis in 63 participants (126 nuclei total). The off-line automated peak detection algorithm identified 71 (56.3%) peaks in 45 patients (71.4%) with similar peak detection between STN and GPi (53/94, 56.4%) peaks in the STN and 18/32, 56.3% peaks in the GPi) (Fig. 2). The peaks were most commonly detected in the high-beta range for both STN and GPi. Although the low-beta peaks were slightly less common, the power of the grand average peak was greatest among the three bands at a frequency of 18.6 ± 6.05 Hz with a range of 9.8–29.3 Hz.

### Adaptive DBS technical performance in and out of clinic

**Fig. 3:**
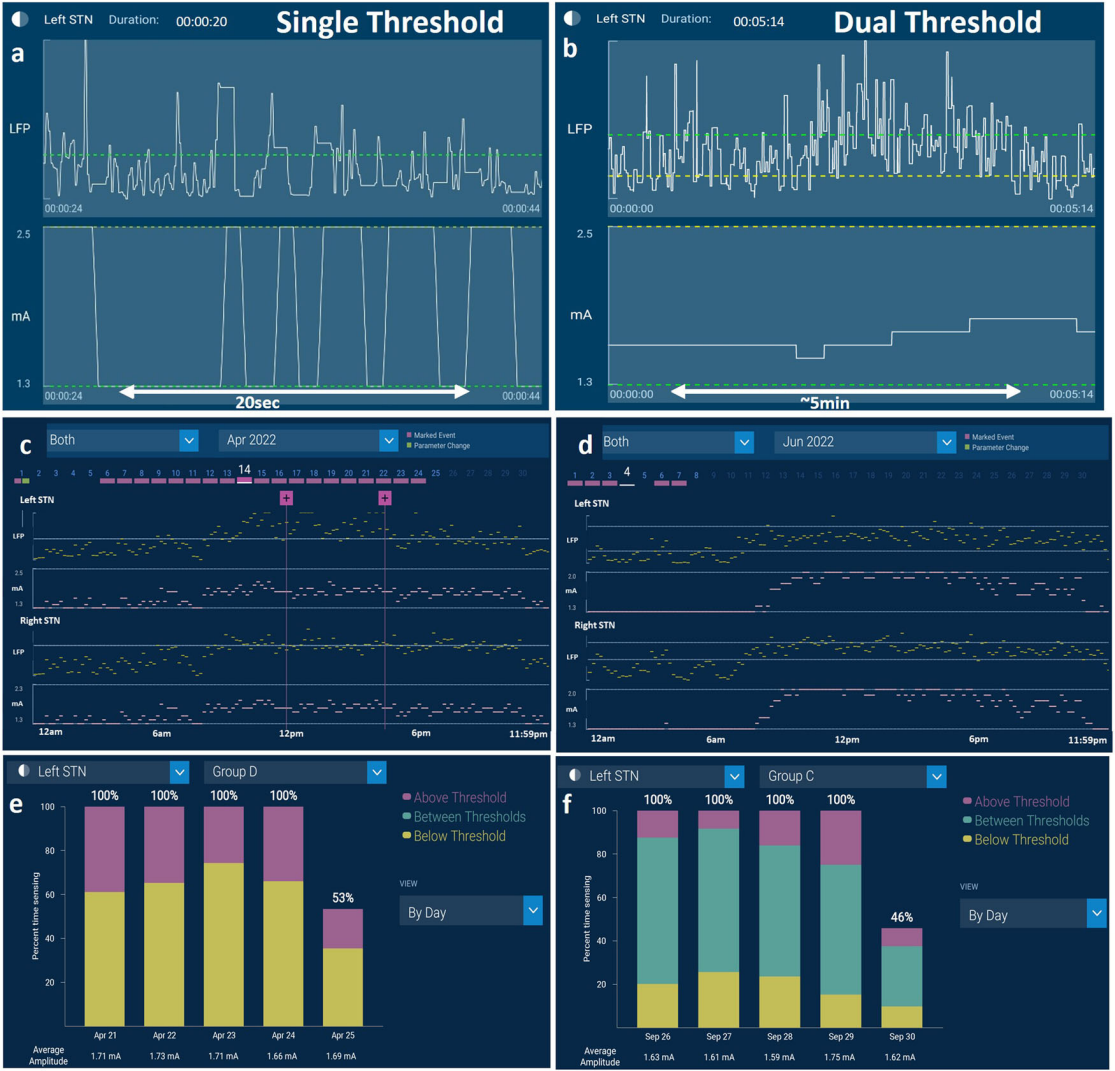
Example of LFP signal visualization during aDBS delivery. BrainSense streaming feature enables visualization of stimulation amplitude adjustment during LFP signal fluctuation above or below LFP thresholds for single threshold (*t* = 20 s) (**a**) and dual threshold (*t* = 5 min) (**b**) mode aDBS; BrainSense Timeline feature demonstrating a 24-h interval of LFP signal fluctuation (yellow) and aDBS stimulation amplitude (pink) for single threshold (**c**) and dual threshold (**d**) mode aDBS. Note the LFP suppression during night hours. Chronic LFP chart illustrating time within threshold for single threshold (**e**) and dual threshold (**f**) mode aDBS. Last day of data in panels (**e**) and (**f**) only contain partial data from 53% and 46% of the day, respectively. Data extracted from a single participant in the ADAPT-PD trial.

An example from one STN participant’s aDBS performance is presented (Fig. 3). In-clinic BrainSense Streaming (Fig. 3a, b) was used to confirm the adaptation of stimulation during task-performance. The difference in therapy delivery is observable in this view: the single threshold mode adjusted stimulation amplitude over 250 ms between the upper and lower stimulation limits set by the clinician, whereas the dual threshold mode adjusted stimulation amplitudes more slowly (increasing amplitude over 2.5 min and decreasing amplitude over 5 min) and each adjustment was incremental rather than ramping fully up and down between the stimulation limits, as seen for single threshold. The BrainSense Timeline views (Fig. 3c, d) demonstrate examples of a 24-h interval of LFP activity (in 10 min averaged bins, yellow data points) and changes in stimulation amplitude (pink), bilaterally, during single (Fig. 3c) and dual (Fig. 3d) threshold aDBS. A clear circadian rhythm can be observed in this view, where averaged alpha-beta band power was lower overnight. Correspondingly, DBS amplitude was lower during the night compared to during the day when alpha-beta power tended to be higher, as well as the general behavior of the two aDBS modes to differ in the variability of the stimulation amplitude delivered both at night and during the day (e.g., more stable stimulation amplitude delivered with dual threshold mode at night). A daily average of the percent time that LFP power was below, between, or above threshold is shown in Fig. 3e, f, and was available for clinicians to assess a day-by-day breakdown of LFP power behavior.

## Discussion

The ADAPT-PD clinical trial was designed to meet evidence needs as a pivotal trial of aDBS for people with PD while still being the first moderate-sized clinical study using aDBS out of clinic, and in combination with directional leads, intended to deliver DBS both *when* and *where* it is needed most. The motivation for this single-blind, randomized crossover clinical trial design was to lay the foundation for aDBS use in a real-world clinical environment with the prioritization of developing evidence for safety and effectiveness that will be an integral step towards making aDBS commercially available.

Enrollment data from the ADAPT-PD trial indicate that an LFP signal sufficient for aDBS programming was present in 84% of patients, regardless of PD clinical subtype, with similar detection rates between STN and GPi, and that medication had a relatively small impact on LFP detection rates (6.7% higher detection rate off medication). Consequently, detection of an LFP signal may be sufficient for aDBS programming in the large majority of patients without necessitating medication withdrawal. One limitation to the reported LFP detection rate is that some study physicians pre-screened participants for LFP peak presence, leading to a potentially inflated LFP detection rate. However, the estimate of peak detection (~84%) is closely aligned with existing literature suggesting a detection rate of 78–95% in the off-medication state^10,21,38,39^. In addition, the majority of patients enrolled had Medtronic 3389/3387 legacy leads. It is expected that it will be easier to detect LFP peaks in patients implanted with SenSight leads, which were designed for sensing LFP signals and suffer less contamination from external noise such as ECG artifact. Peaks chosen by clinicians in the ADAPT-PD trial were distributed among alpha (8–12 Hz), low-beta (13–20 Hz), and high-beta (20–30 Hz) bands, the majority of which were in the beta band in both the STN and GPi. There was a wide variation among peak power from 1.2 µVp (the lowest acceptable power to run aDBS) to almost 9 µVp. An interesting observation in the distribution of LFPs is that alpha peaks occurred in the alpha range at a higher frequency in the GPi (59%) compared to the STN (20%) when off medication. This finding warrants continued study to understand what clinical significance this differential distribution of low-frequency LFP signals may have.

Two modes or algorithms for aDBS are used in the ADAPT-PD trial, both of which have been shown previously to provide efficacious therapy in smaller, single-center studies^27,30^. The Single Threshold mode was designed to adapt stimulation amplitude rapidly between lower and upper stimulation limits in 250 ms. If LFP power remains above the LFP threshold, single threshold DBS intensity remains at the upper limit and if power remains below the LFP threshold, single threshold DBS intensity remains at the lower stimulation limit. This results in a trapezoidal pattern of aDBS ramping (Fig. 3a). In the original single threshold study by Little et al., it had been shown that aDBS effective stimulation was associated with active stimulation at the high level less than 50% of the time in the medication off state. In addition, patient perception of aDBS (through e.g., paresthesia) was related to the rate of ramping up or down of DBS amplitude^27^. These features are carefully monitored during the ADAPT-PD aDBS Setup and Adjustment Phase. One technical difference between the current study and original adaptive studies is the hemispheric use of bilateral single threshold^36^. In the ADAPT-PD study, the LFP signal crossing the threshold in one hemisphere results in bilateral control of stimulation amplitude, with the lower stimulation limit set to the lowest constant amplitude at which symptoms are controlled per clinician determination. Another difference between the seminal single threshold literature and the ADAPT-PD trial is that single threshold aDBS was enabled early after implant in Little et al., and after stable chronic cDBS in the ADAPT-PD trial.

Dual threshold aDBS adjusts DBS amplitude more incrementally based on LFP power: if LFP power is above the upper LFP threshold, stimulation amplitude is increased; if LFP power is in between the upper and lower thresholds then stimulation amplitude remains constant; and if LFP power is below the lower LFP threshold then stimulation amplitude decreases. This results in smaller amplitudes of adjustment, each of which did not span the range of the aDBS amplitude window (Fig. 3b). In the ADAPT-PD trial the rates of increasing and decreasing of stimulation amplitude in the dual threshold mode are much slower than in the single threshold mode (2.5 min up and 5 min down for dual threshold compared to 250 ms up or down for single threshold). The timescales of the single and dual threshold adjustments in Fig. 3a, b are different to reflect the rapid and slow adaptions, respectively. In prior studies, aDBS in both modes was allowed to decrease stimulation amplitude to zero^27,30^ but this was shown in at least one study to occasionally result in sub-therapeutic therapy, resulting in a return of PD symptoms^30^. Consequently, in the ADAPT-PD study, the lower stimulation limit is individually chosen as that at which there was still acceptable therapeutic improvement.

The two modes were chosen in the ADAPT-PD trial to allow flexibility in programming to meet patient needs. For the single threshold mode, a rapid rate of increase of stimulation amplitude was theorized to suppress long beta burst durations, which have been linked to symptomatic states and modulates how often adaptive stimulation ramps stimulation according to medication state^36,40,41^; in contrast, the dual threshold mode was theorized to adjust stimulation along the timescale of medication influences on beta band power^15,17^, where beta band power decreases with the onset of medication and increases as medication wears off. The theory of slowly adapting aDBS was that it would allow beta band power to remain within a certain therapeutic window defined by the therapeutic window of DBS amplitude rather than wider fluctuations with traditional cDBS plus medication. In dual threshold the onset time requirements are less. The onset duration is programmable by the clinician to meet patient needs in a range of 1.2–2 s. The design drivers in setting onset for this algorithm was to ensure no false detection of stimulation changes and return to sensing with this slowly adapting algorithm. The differing performance of the two aDBS modes provide patients and clinicians with flexibility to personalize aDBS therapy to patient neurophysiology and clinical need. For instance, if a patient was experiencing uncomfortable motor fluctuations on cDBS plus medication, the physician may want to start with a trial of dual threshold aDBS. Whereas if a patient was experiencing good control in cDBS, but experiences challenges with narrow therapeutic window of reduced efficacy at top level of stimulation due to side effects (e.g., speech), single threshold could be leveraged by the physician^36^. Another potential use for dual or single threshold could be with slow unilateral fluctuations where only one hemisphere’s LFP power could be used to smooth those fluctuations.

This manuscript has detailed the ADAPT-PD protocol, in which people with PD experienced real-world aDBS therapy for an extended period of time. It has shown that LFP peaks with an adequate power to track ON aDBS were identifiable in the majority of patients both off and on medication and spanned the 8–30 Hz frequency range, with the majority in the beta band. Two different modes of aDBS were used with different timescales of aDBS amplitude adjustment that reflect different therapeutic goals and allow flexibility in the choice of aDBS for individual patients in the pursuit of personalized neuromodulation. Knowledge gained from the results of the ADAPT-PD trial will advance the understanding of: (1) safety of chronic aDBS out of clinic, (2) aDBS programming workflow and feasibility, (3) clinical advantages of aDBS compared to cDBS, (4) patient preference and experience, (5) which patients may benefit the most from aDBS, and (6) effects of combining directional and adaptive stimulation for the first time. These results will provide the groundwork to clinically implement aDBS for PD and potentially support applications in other movement and neurological disorders that may also benefit from adaptive stimulation^23,42^.

## Methods

**Fig. 4:**
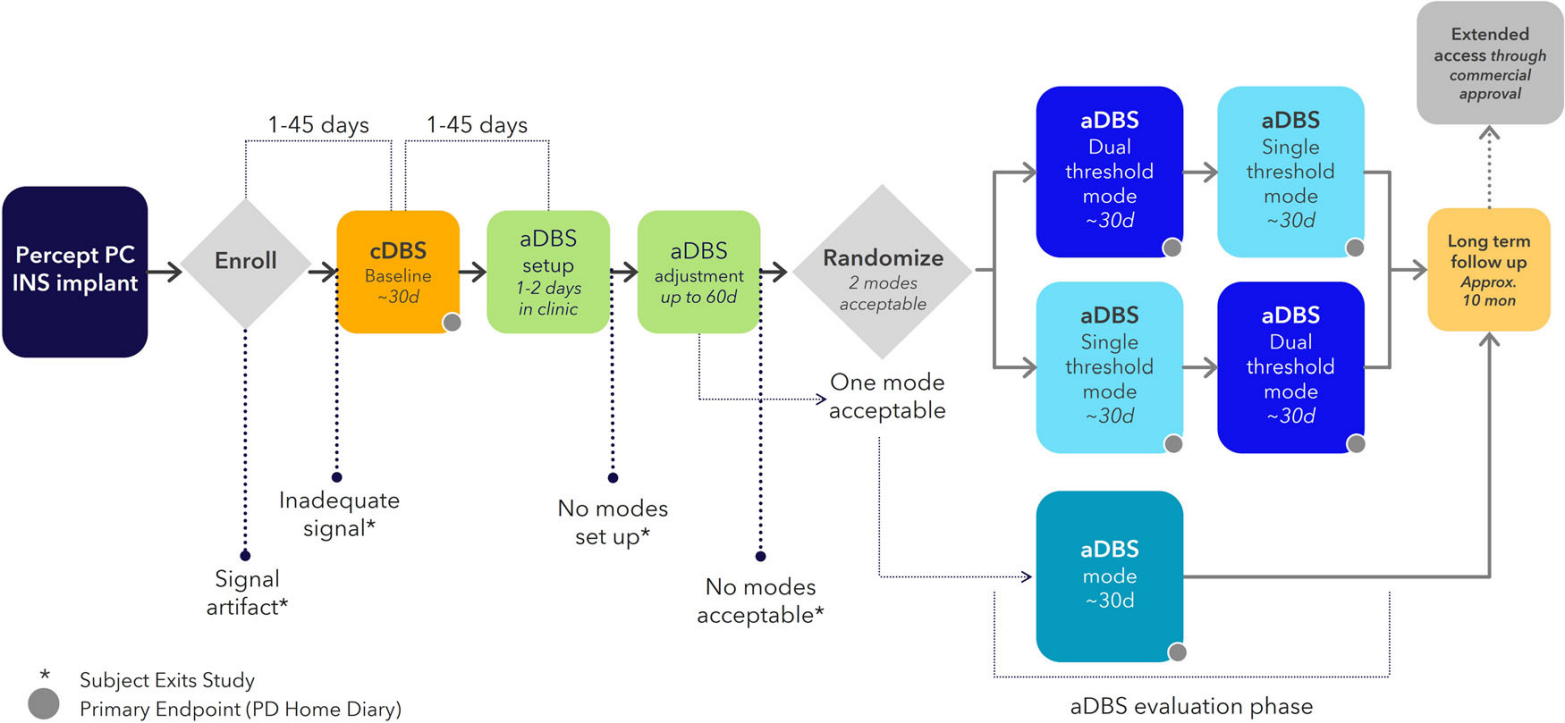
ADAPT-PD trial visit schedule. The study schedule is formed by four general phases: the cDBS Baseline (orange), aDBS Setup and Adjustment (green), aDBS Evaluation (blue), and Long-term Follow-up (gold). Extended Access after Long-term Follow-Up was optional (gray). Participants exited the study due to signal artifact, inadequate LFP signal, or if no aDBS modes were found to be acceptable/tolerated.

The ADAPT-PD trial is a global, multicenter, prospective, single-blind, randomized crossover clinical investigation evaluating the safety and effectiveness of aDBS for PD. The study includes two cohorts, one without directional stimulation (Primary Cohort) and one with directional stimulation (Directional Stimulation Cohort). The study consists of four phases following enrollment: cDBS Baseline, aDBS Setup and Adjustment, aDBS Evaluation, and Long-term Follow-up (Fig. 4). Following aDBS Setup and Adjustment, an Evaluation Phase (Fig. 4, blue section) is completed. The Evaluation phase includes a randomized crossover to aDBS single or dual threshold mode for participants that had an acceptable response to both aDBS modes (see “Methods” for mode description). If only one aDBS mode is acceptable, then only that mode is evaluated in the Evaluation Phase. An optional Extended Access Phase is offered to participants who wish to continue receiving aDBS therapy after Long-term Follow-Up. The ADAPT-PD trial is registered on ClinicalTrials.gov (NCT04547712).

### Population

Individuals eligible for enrollment have stable STN or GPi DBS therapy and medication for idiopathic PD with a previously implanted commercial DBS system capable of sensing LFPs (Medtronic, Percept™ PC). Participants enrolled in either the Primary Cohort (Medtronic legacy or Medtronic SenSight^TM^ leads with omni-directional stimulation) or Directional Cohort using SenSight leads capable of delivering directional stimulation (see Supplemental Table 1, inclusion criteria for more information). Further inclusion criteria include responsiveness to DBS in the opinion of the investigator based on each investigator’s standard of care in their DBS practice (including but not limited to MDS-UPDRS score improvements on DBS). In addition, configuration to monopolar or dual monopolar stimulation with a peak alpha-beta frequency (8–30 Hz) with amplitude ≥1.2 µVp detected on either left and/or right DBS leads while OFF stimulation/off medication (see Sup. Table 1 for full eligibility criteria). Participants complete screening for suicidality with the Columbia-Suicide Severity Rating Scale. Patients with unapproved hardware in the brain or significant signal artifact on all 6 bipolar sensing pathways are excluded (see Goyal et al, Fig. 1d for example of sensing pathways^43^). Artifacts are detected by software on the clinician tablet designed to identify movement and ECG (Electrocardiogram) artifacts if they occur. The study was approved by the Institutional Review Board or Ethics Committee of each participating center and conformed to the Declaration of Helsinki, Good Clinical Practice Guidelines, and ISO 14155, 2020. All participants provide written informed consent. An independent Data Monitoring Committee advises Medtronic regarding the safety of study subjects and the validity and scientific merit of the study.

### Study endpoints and outcomes

The primary endpoint of the ADAPT-PD clinical trial evaluates the proportion of subjects where aDBS and cDBS have similar “On” time without troublesome dyskinesia. “On” time is calculated from the Parkinson’s Disease Home Diary (PD Home Diary) during a two-week window (3-day diary) prior to the cDBS Baseline visit and the aDBS Evaluation visits (single and/or dual mode). The secondary endpoint is the total electrical energy delivered (TEED) calculated by established criteria^44^. Additional outcomes include: the Movement Disorders Society - Unified Parkinson’s Disease Rating Scale (MDS-UPDRS, part II, III, IV), Voice Handicap Scale (VHI), European Quality of Life – 5 Dimensions, version 5 L (EQ-5D-5L), Parkinson’s Disease Questionnaire (PDQ-39), Parkinson’s Disease Sleep Scale 2 (PDSS-2), Medtronic-developed aDBS Global Impression of Change (GIC) and objective movement data collected via a wearable watch over 6 days during each DBS Evaluation Phase (PKG, Global Kinetics). The PKG watch is worn by the patient for 6 days during the 2-week interval leading up to an Evaluation visit. Patient preference and satisfaction for DBS mode (cDBS, aDBS single, aDBS dual) is collected via a Medtronic-developed questionnaire. All outcomes are collected during cDBS Baseline Phase and aDBS Evaluation Phase (for each mode programmed). Safety assessments are performed to characterize stimulation-related adverse events during the aDBS Evaluation and the cDBS Baseline Phases. Adverse events and device deficiencies are monitored throughout the study. Participants are instructed to pause aDBS at any time (enabling their previously established cDBS settings) if they feel that aDBS is not working well for them, and to notify the clinician for adjustment and completion of an appropriate adverse event documentation.

### Device description

The commercially approved Medtronic Percept™ PC DBS device is capable of simultaneously delivering electrical stimulation therapy and recording LFP activity through the same DBS leads implanted into the brain^43,45,46^. The Percept BrainSense™ Survey feature presents power spectral density plots from 30 s differential recordings across the DBS lead ring and segmented contacts. The BrainSense Signal Test reports the largest signal peak that is >1.1 µVp from the alpha, beta, or gamma frequency range across the three differential ring levels. BrainSense Streaming feature displays LFP power in a selected 5 Hz-wide frequency band around the chosen peak frequency in real time during stimulation parameter changes. During streaming, broad-band (0–100 Hz) time domain data is captured for off-line analysis but not displayed on the programming tablet interface. The BrainSense Timeline feature allows a 5 Hz-wide frequency band of interest to be recorded chronically outside of the clinic and stored as 10 min averages for up to 60 days. The Event Capture feature allows a patient to trigger a 30 s power spectrum recording (0–100 Hz) during up to four unique clinically defined events (e.g., during periods of dyskinesia).

**Fig. 5:**
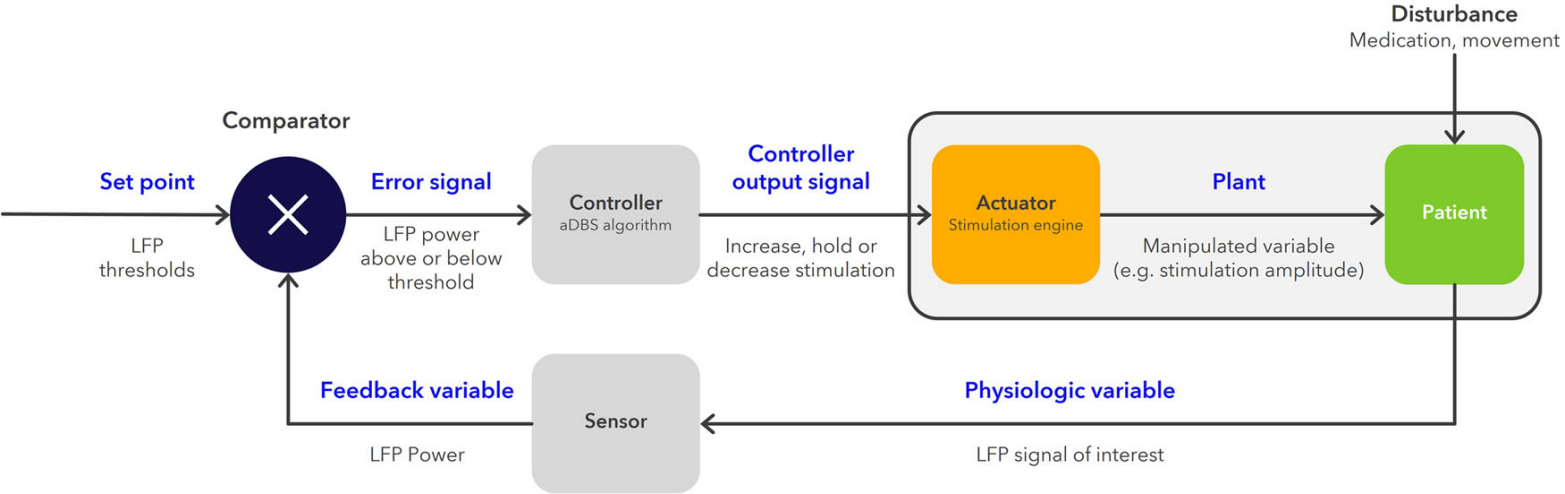
Adaptive DBS as a Physiological Closed-Loop Control (PCLC) technology. Adapted from Technical Considerations for Medical Devices with Physiologic Closed-Loop Control Technology - Final Guidance for Industry and Food and Drug Administration Staff^47^. In the ADAPT-PD trial, the Percept device PCLC operates with the LFP power thresholds serving as the system Set Points. The LFP thresholds are the inputs to the Comparator block, which continuously evaluates Feedback variable levels (i.e., LFP signal of interest power) in comparison to the LFP thresholds. The Error signal (LFP control signal above, between, or below threshold) triggers the Controller (aDBS algorithm) output signal to command the Actuator (Percept Simulation Engine) to adjust stimulation when appropriate.

The Percept device also contains an investigational latent bidirectional functionality that can be unlocked with software for delivering LFP-controlled aDBS algorithms. With the aDBS functionality activated, the Percept device can operate as a Physiological Closed-Loop Controlled (PCLC) device per guidance from the United States Food and Drug Administration^47^ (Fig. 5). In the Percept PC aDBS system, the Actuator is the stimulation engine which controls the stimulation amplitude delivered to the patient. In addition, another input Disturbance signal to the Plant exists. For aDBS these Disturbance sources could be but are not limited to medication or movement artifact and can impact the patient and the Physiologic variable (LFP signal of interest). The LFP signal of interest is a time domain signal sampled at 250 Hz by the Sensor block^45^. The Feedback variable (LFP Power integrated over a 5 Hz band) is delivered back to the Comparator to complete the PCLC loop. The output of the Comparator is an assessment of whether the LFP power is above, below, or between thresholds. The Controller (aDBS algorithm) processes the comparator output and sends the Control signal to the stimulation engine. The stimulation engine then controls the stimulation amplitude delivered to the patient when the LFP Power migrates beyond the Set Point (LFP thresholds). The ADAPT-PD trial evaluates two aDBS modes: single and dual-threshold aDBS. For both modes, the upper and lower stimulation limits correspond to minimum and maximum safe stimulation amplitudes that provide adequate therapy and are below the amplitude that results in stimulation induced adverse effects, respectively. Stimulation limits are determined by the clinician in a patient-specific manner. The LFP brain signal is first sensed and processed by the Sensor block (Percept Sense Chip)^45^, and the Feedback variable (e.g., patient-specific LFP power) is fed back to the Comparator, which calculates an Error Signal relative to the Set Point and when appropriate, sends stimulation commands to the Controller to complete the PCLC loop. Another potential input to the Percept Actuator is a Disturbance signal, which may include movement or cardiac artifacts that can inappropriately impact the Error signal. Movement and cardiac artifacts are filtered by the Percept system using signal averaging and onset timers to minimize improper adaptation. In addition, slow adapting ramp rates for the dual threshold algorithm further filter movement artifacts. Finally, artifact detection algorithms are run during the aDBS Setup phase and stimulation titration with LFP streaming performed to insure the LFP control signal is properly responsive to stimulation.

### Single threshold mode

**Fig. 6:**
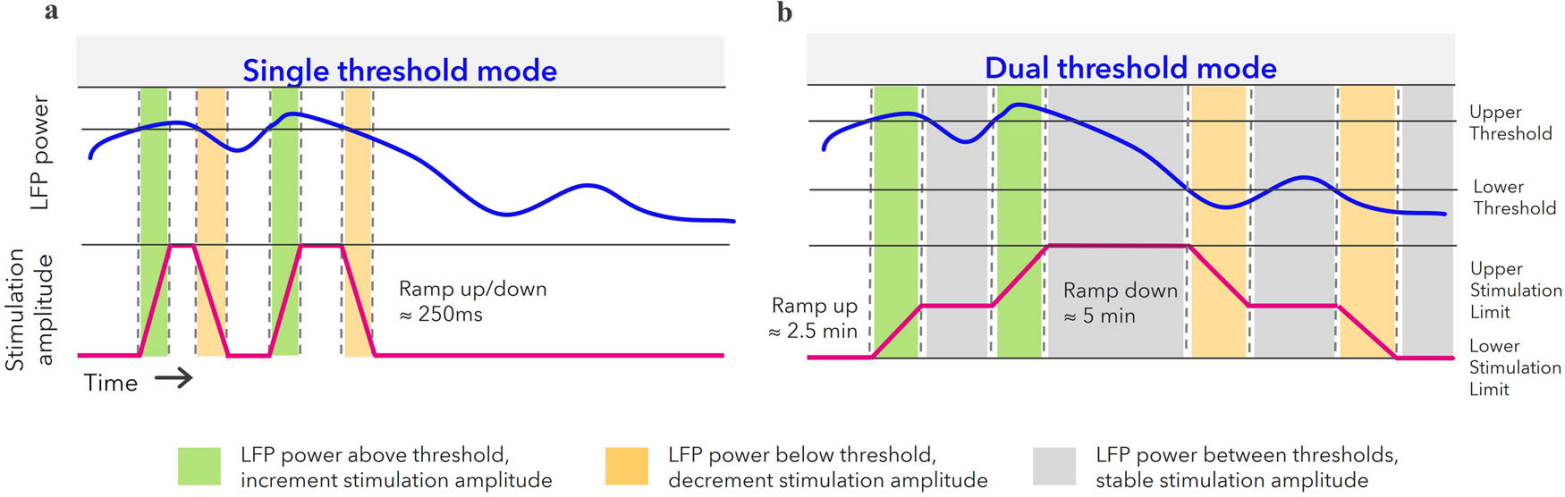
Adaptive deep brain stimulation algorithmic modes. Single threshold mode (**a**) adapts stimulation amplitude up or down with a fast ramp (100’s of milliseconds) when the LFP control signal (i.e., LFP power) is above or below a pre-established threshold limit. Dual threshold mode (**b**) adapts stimulation with a slow ramp (min) when the LFP power of the control signal is above or below pre-established thresholds.

The single threshold algorithm uses fast (sub-second) rates of increasing or decreasing stimulation amplitudes (ramp rates) in response to whether alpha-beta power is above or below a single LFP power threshold. The single threshold algorithm was designed to provide a method for rapid adaptation that may in theory modulate stimulation amplitude up during high amplitude and long duration beta bursts (>500 ms) associated with low medication states and/or increased PD motor symptoms^40^. The single threshold algorithm directs the neurostimulator to increase stimulation amplitude to the upper stimulation limit when LFP band power exceeds the threshold and to decrease stimulation amplitude toward the lower stimulation limit when the LFP power falls below the threshold (Fig. 6a). The LFP band power is calculated by the Percept PC device by taking the fast Fourier transform of the time series data and averaging it every 100 ms. The time above or below threshold needs to exceed an onset duration before changing stimulation. The onset duration is programmable by the clinician to meet patient needs in a range 200–500 ms. The onset time range is intended to ensure the detection is fast enough to identify long duration alpha-beta signals >500 ms and to avoid false detecting stimulation changes (along with the blanking duration default 550 ms following stimulation changes). LFP power with amplitudes above the LFP threshold for longer than the onset duration trigger stimulation amplitude changes with proper control of the detection blanking time, stimulation ramp time and detection onset times. For single threshold mode in a bilaterally implanted patient, if the LFP power crosses the LFP threshold within one hemisphere, a change in stimulation is directed to both hemispheres. This was a design decision made for the implantable Percept system to avoid blanking the opposite hemisphere and under stimulating when detection occurred in 1 hemisphere. In addition, for single threshold mode, if only one hemisphere has a sufficient LFP signal, aDBS is configured only in that hemisphere, while leaving cDBS on in the hemisphere with an inadequate signal to program aDBS.

### Dual threshold mode

The dual threshold aDBS algorithm operates with relatively slower ramp rates and provides a method to adapt stimulation on a slow minute-to-minute timescale that may optimally adjust to medication wash-in timing and mitigate motor fluctuations and dyskinesias^30^. The dual threshold algorithm directs the neurostimulator to increase stimulation amplitude when the power of the LFP reaches above the upper threshold, and to decrease stimulation amplitude when the power of the LFP drops below the lower threshold, but to hold the stimulation amplitude when the power of the LFP remains between the thresholds (Fig. 6b). For dual threshold mode, if only one hemisphere has a sufficient LFP signal, aDBS could be configured to control stimulation amplitude in both hemispheres using the single viable LFP signal.

### Study phases and visits

**Fig. 7:**
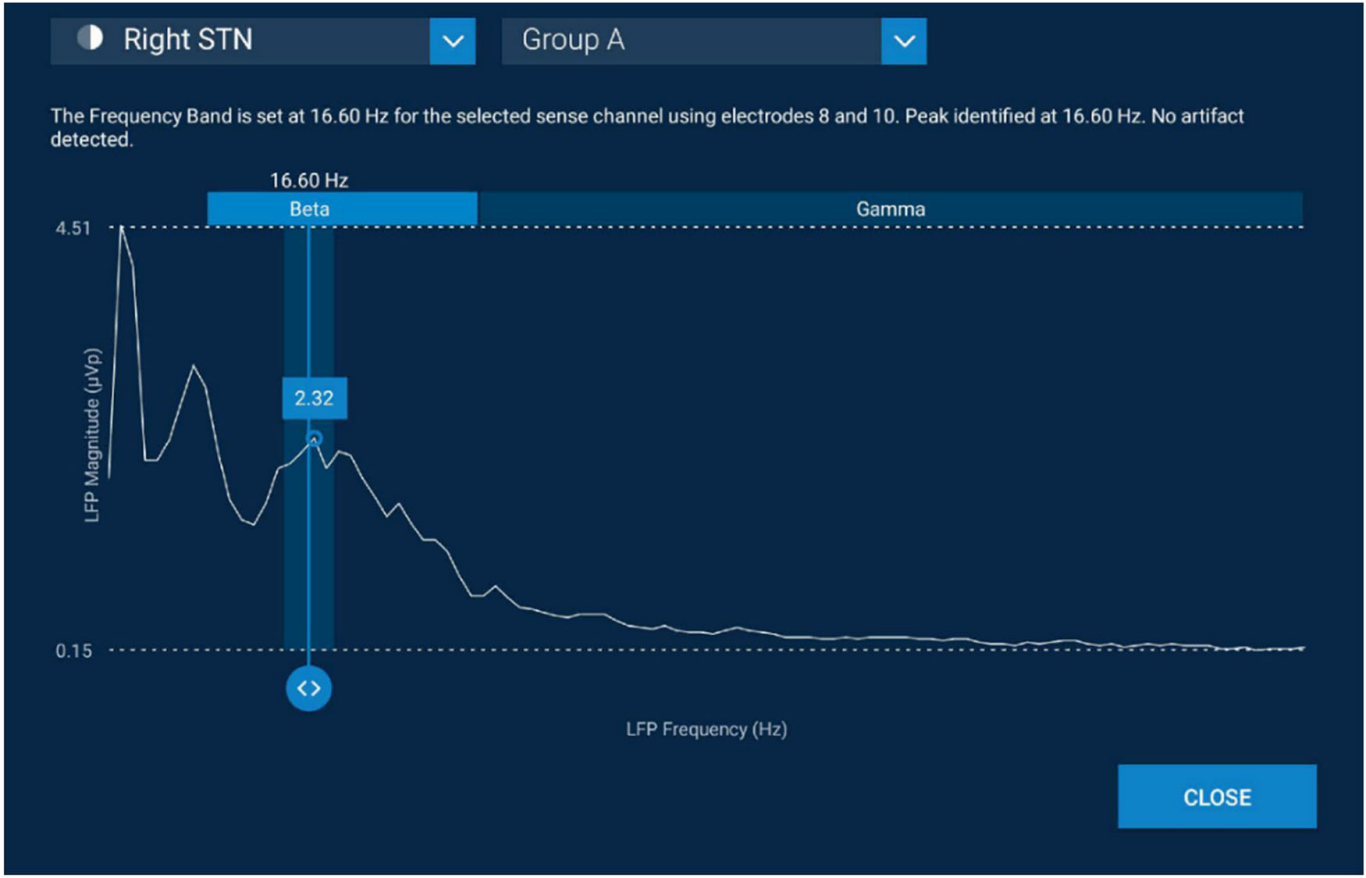
Example LFP peak detected by the Percept BrainSense Signal Test. Example power spectral density plot (PSD) shows 16.6 Hz LFP beta signal with 2.32 µVp that was used as the aDBS control signal for the Right STN of a study participant.

At the initial Enrollment visit (Fig. 4, gray diamond), consent, baseline demographic, medical, and device information are collected. Patients arrive ON cDBS/on medication at this visit. Stimulation was turned OFF and after ~15 min the LFP signals are acquired using the Percept device BrainSense^TM^ Signal Test (Fig. 7) to confirm at least one sensing pathway without artifact is detected, indicating the patient has a viable LFP signal for adaptive DBS setup. The clinician documents the peak frequency and amplitude observed and an LFP file is saved to the device for analysis.

The cDBS Baseline Phase (Fig. 4, orange square) begins with an LFP screening via BrainSense^TM^ Signal Test (Fig. 7) 1–45 days after enrollment. At the LFP screening, the participant arrives off medication and ON cDBS. The cDBS is then turned OFF for 15 min, after which the MDS-UPDRS III and LFP signals are collected OFF stimulation/off medication via BrainSense signal test. If the algorithm identifies a potential artifact, the clinician is then permitted to visually inspect the power spectral density plot and manually identify a potential LFP peak within the 8–30 Hz range with adequate signal strength. Patients with artifact on all channels precluding visualization of an LFP signal are exited from the study. Only subjects with at least one hemisphere with adequate signal to program aDBS moved forward with the study (i.e., LFP peak frequency in the alpha-beta range (8–30 Hz) and peak power was ≥1.2 µVp). The cDBS Baseline Phase is then completed over a 30-day period during which the subjects are monitored on their pre-study clinical cDBS settings with passive sensing turned on (BrainSense Timeline and Event Capture). The cDBS Baseline visit, performed at the end of this 30-day period, consists of collecting all outcomes (see section Study Endpoints and Outcomes).

At the aDBS Setup visit (1–2 days; Fig. 4, green square), participants are evaluated off medication and the investigational aDBS feature is unlocked to program aDBS using an investigational programming tablet. BrainSense Signal Test and Survey (Fig. 7) is used to review the 8–30 Hz LFP frequency range, whereby a 5 Hz band around the largest identified peak is initially chosen as the aDBS control signal for each brain hemisphere where applicable. The aDBS program for each participant is set up by the study clinician separately for the single threshold and dual threshold modes using a defined programming workflow (Table 3). Initially safe and efficacious upper and lower stimulation limits (respectively) are determined based on a clinical assessment of therapeutic efficacy and lack of adverse effect, which are then used in combination to set the aDBS algorithm LFP thresholds. The BrainSense Streaming feature (Fig. 3a, b) is then used to evaluate the clinical and LFP signal impacts of stimulation amplitude adjustments between these limits and using the chosen threshold(s). A similar procedure is performed when the participant is on medication, usually the following day. Following the aDBS Setup and during the aDBS Adjustment Phases, the BrainSense Timeline (Fig. 3c, d) is utilized to record, monitor, and visualize the aDBS LFP control signal to ensure that the Percept device is properly triggering stimulation amplitude adjustments when the LFP power crosses the LFP thresholds.

**Table 3:** Adaptive DBS programming steps

Programming step	Dual threshold mode	Single threshold mode
1. Determine upper stimulation limit *off Medication*	DBS stimulation set to the highest safe constant amplitude at which symptoms are controlled and stimulation-related side effects are minimized per clinician determination.	
1. 2. Determine lower stimulation limit *on Medication*	DBS stimulation set to the lowest constant amplitude at which symptoms are controlled per clinician determination.	
3. Determine upper LFP threshold *off Medication*	DBS stimulation is set to the lower stimulation limit. The average LFP level over a 30 s epoch is set as the upper LFP threshold.	DBS stimulation is set to 0 mA. The average LFP level over a 30 s epoch is recorded as the upper LFP threshold.
4. Determine lower LFP threshold *off Medication*	DBS stimulation is set to the upper stimulation limit. The average LFP level over a 30 s epoch is set as the lower LFP threshold.	Next stimulation is set to the upper stimulation limit. Another 30 s average LFP is calculated as the lower LFP threshold. Finally, the single threshold mode default is set at 75% of the way between upper threshold and lower threshold.
5. Optimize Ramp Time	Default ramp time of 2.5 min upwards and 5 min downwards. Ramp time optimization was completed using a 5-min test interval with repeated wrist flexion and voluntary movements to trigger LFP modulation and simultaneous DBS modulation. Ramp time was allowed to range from 1 to 10 min upwards, and 1 to 10 min downwards per clinician determination.	Default ramp time of 250 ms. Ramp time optimization was completed using a 5-min test interval with repeated wrist flexion and voluntary movements to trigger LFP modulation and simultaneous aDBS modulation.
6. Confirm aDBS performance *after medication wash-in*	The lower stimulation limit was adjusted to provide acceptable therapy without symptom breakthrough. aDBS performance was confirmed with a clinical examination per standard of care clinical assessment and the MDS-UPDRS part III.	A lower stimulation limit was adjusted to provide acceptable therapy without symptom breakthrough. The single threshold was adjusted to achieve therapeutic stimulation “On” time without paresthesia. aDBS performance was confirmed with a clinical examination per a standard of care clinical assessment and the MDS-UPDRS part III.

*aDBS* adaptive deep brain stimulation, *LFP* local field potential, *MDS-UPDRS* Movement Disorder Society Unified Parkinson’s Disease Rating Scale.

The purpose of the aDBS Adjustment Phase is to personalize and optimize aDBS with a combination of in-clinic and at-home use. This phase can last up to 2 months, during which the clinician assesses each participant’s response to aDBS while adjusting and optimizing stimulation amplitude ramping and limits and the LFP thresholds during unscheduled clinical visits. Adjustments are determined by the clinician using in-clinic assessments as well as BrainSense Timeline (Fig. 3c, d) and estimated time below (Fig. 3e) or between (Fig. 3f) thresholds depending on which mode aDBS is being adjusted. During this phase, the participants remain blinded to the aDBS mode being adjusted. At the completion of this phase, clinician and participant satisfaction with the aDBS settings are assessed, and none, one or both modes are determined to be acceptable using a modified Global Impression of Change score of ≤8 as a threshold for moving on to the Randomization and/or Evaluation Phase.

Participants determined to have an acceptable response to both aDBS modes are randomized to a crossover period for two 30-day aDBS Evaluation periods (Fig. 4, blue rectangles), whereas those with only one acceptable aDBS mode complete a single 30-day Evaluation period. Any participant who does not have an acceptable response to either aDBS mode exits the study. All Evaluation Phase endpoints and outcome measures are collected with the participant on their programmed aDBS settings. Participants remain blind to the mode chosen.

After completion of the aDBS Evaluation Phase, participants who prefer cDBS exit the study. Participants who prefer aDBS enter a Long-term Follow-up Phase programmed on their preferred aDBS mode for ~10 months, during which four scheduled visits are completed. Following completion of the Long-term Follow-up Phase, optional extended access to aDBS are provided with follow-up visits every 6 months.

### Statistical methods

The purpose of the ADAPT-PD trial is to demonstrate the safety and effectiveness of aDBS in patients with PD. The primary endpoint selected is a performance goal demonstrating that aDBS is effective in the majority of the study participants during the Evaluation Phase. The performance goal is that >50% of the study participants have similar “On” time without troublesome dyskinesia between cDBS and aDBS. The sample size was calculated using a binomial distribution for a one-sided exact test (alpha = 0.0125), alternative hypothesis of 85%, resulting in a minimum of 36 subjects to achieve at least 90% power to reject a performance goal of 50%. Single Threshold mode and Dual Threshold mode will be evaluated separately using a Bonferroni corrected alpha (0.0125) for the respective hypothesis (0.025/2). SAS® version 9.4 was used for all statistical analyses.

Primary and secondary objectives will be analyzed using the intention-to-treat (ITT) principle in the Full Analysis Set (FAS): participants who initiate the aDBS Evaluation Phase for each subject that was randomized and the programmed treatment assignment for those subjects that are only configured to one aDBS mode (dual or single threshold). The performance goal threshold for the primary objective is determined from the standard deviation of the difference between aDBS and cDBS and set at the hours of “On” time without troublesome dyskinesia for aDBS is no worse than one standard deviation less than cDBS. Missing data will be addressed using multiple imputation. The secondary objective evaluates the difference in TEED between aDBS and cDBS for each mode for subjects in the FAS. Carry-over effects during the Evaluation Phase for the primary and secondary outcome will be evaluated.

Demographics and patient characteristics will be calculated with summary statistics in the Primary Cohort only. PD subtype is calculated using the OFF stimulation/off medication MDS-UPDRS part III score collected at the LFP screening and scoring criteria will be adapted from previously established methods^48^. LFP data will be analyzed for the Enrollment (on medication) and LFP Screening (off medication) visits with summary statistics. Statistical results will be calculated with SAS software (version 9.4 or higher). The LFP frequency band categorization is alpha (8–12), low-beta (13–20), and high-beta (21–30).

## Supplementary information

Supplementary consortia list and supplemental table.

## Data Availability

A minimal dataset will be available from the corresponding author upon reasonable request. The study sponsor (Medtronic) will evaluate on a case-by-case basis whether there is an opportunity to share clinical trial data with qualified scientific or medical researchers, consistent with the associated informed consent and applicable laws and regulations. Individual participant data will not be shared and secondary use will not be permitted due to global data privacy law restrictions.

## Code availability

All data analysis leveraged for this study by the Percept PC implantable Neuro-stimulator system to collect, analyze and process the data can be accessed in the Medtronic manuals located here: https://manuals.medtronic.com/manuals/. Figure 1 was created with GraphPad Prism 9. Figure 2 was created with Python 3.11.

## References

[1] Limousin, P. & Foltynie, T. Long-term outcomes of deep brain stimulation in Parkinson disease. *Nat. Rev. Neurol.* **15**, 234–242 (2019). 30778210 10.1038/s41582-019-0145-9

[2] Fasano, A., Aquino, C. C., Krauss, J. K., Honey, C. R. & Bloem, B. R. Axial disability and deep brain stimulation in patients with Parkinson disease. *Nat. Rev. Neurol.* **11**, 98–110 (2015). 25582445 10.1038/nrneurol.2014.252

[3] Fox, S. H. et al. International Parkinson and movement disorder society evidence-based medicine review: Update on treatments for the motor symptoms of Parkinson’s disease. *Mov. Disord.* **33**, 1248–1266 (2018). 29570866 10.1002/mds.27372

[4] Schuepbach, W. M. M. et al. Neurostimulation for Parkinson’s disease with early motor complications. *N. Engl. J. Med* **368**, 610–622 (2013). 23406026 10.1056/NEJMoa1205158

[5] Weaver, F. M. et al. Bilateral deep brain stimulation vs best medical therapy for patients with advanced Parkinson disease: a randomized controlled trial. *JAMA* **301**, 63–73 (2009). 19126811 10.1001/jama.2008.929 PMC2814800

[6] Timmermann, L. et al. Multiple-source current steering in subthalamic nucleus deep brain stimulation for Parkinson’s disease (the VANTAGE study): a non-randomised, prospective, multicentre, open-label study. *Lancet Neurol.* **14**, 693–701 (2015). 26027940 10.1016/S1474-4422(15)00087-3

[7] Vitek, J. L. et al. Subthalamic nucleus deep brain stimulation with a multiple independent constant current-controlled device in Parkinson’s disease (INTREPID): a multicentre, double-blind, randomised, sham-controlled study. *Lancet Neurol.* **19**, 491–501 (2020). 32470421 10.1016/S1474-4422(20)30108-3

[8] Pinter, D. et al. Antiparkinsonian drug reduction after directional versus omnidirectional bilateral subthalamic deep brain stimulation. *Neuromodulation* **26**, 374–381 (2023). 35190245 10.1016/j.neurom.2022.01.006

[9] Bhidayasiri, R. & Truong, D. D. Motor complications in Parkinson disease: clinical manifestations and management. *J. Neurol. Sci.* **266**, 204–215 (2008). 17897677 10.1016/j.jns.2007.08.028

[10] Little, S. & Brown, P. What brain signals are suitable for feedback control of deep brain stimulation in Parkinson’s disease? *Ann. N. Y Acad. Sci.* **1265**, 9–24 (2012). 22830645 10.1111/j.1749-6632.2012.06650.x PMC3495297

[11] Weinberger, M. et al. Beta oscillatory activity in the subthalamic nucleus and its relation to dopaminergic response in Parkinson’s disease. *J. Neurophysiol.* **96**, 3248–3256 (2006). 17005611 10.1152/jn.00697.2006

[12] Kuhn, A. A. et al. Pathological synchronisation in the subthalamic nucleus of patients with Parkinson’s disease relates to both bradykinesia and rigidity. *Exp. Neurol.* **215**, 380–387 (2009). 19070616 10.1016/j.expneurol.2008.11.008

[13] Chen, C. C. et al. Complexity of subthalamic 13–35 Hz oscillatory activity directly correlates with clinical impairment in patients with Parkinson’s disease. *Exp. Neurol.* **224**, 234–240 (2010). 20353774 10.1016/j.expneurol.2010.03.015

[14] Pogosyan, A. et al. Parkinsonian impairment correlates with spatially extensive subthalamic oscillatory synchronization. *Neuroscience* **171**, 245–257 (2010). 20832452 10.1016/j.neuroscience.2010.08.068

[15] Kuhn, A. A., Kupsch, A., Schneider, G.-H. & Brown, P. Reduction in subthalamic 8–35 Hz oscillatory activity correlates with clinical improvement in Parkinson’s disease. *Eur. J. Neurosci.* **23**, 1956–1960 (2006). 16623853 10.1111/j.1460-9568.2006.04717.x

[16] Kuhn, A. A. et al. High-frequency stimulation of the subthalamic nucleus suppresses oscillatory beta activity in patients with Parkinson’s disease in parallel with improvement in motor performance. *J. Neurosci.* **28**, 6165–6173 (2008). 18550758 10.1523/JNEUROSCI.0282-08.2008 PMC6670522

[17] Ray, N. J. et al. Local field potential beta activity in the subthalamic nucleus of patients with Parkinson’s disease is associated with improvements in bradykinesia after dopamine and deep brain stimulation. *Exp. Neurol.* **213**, 108–113 (2008). 18619592 10.1016/j.expneurol.2008.05.008

[18] Feldmann, L. K. et al. Subthalamic beta band suppression reflects effective neuromodulation in chronic recordings. *Eur. J. Neurol.* **28**, 2372–2377 (2021). 33675144 10.1111/ene.14801

[19] Arlotti, M. et al. Monitoring subthalamic oscillations for 24h in a freely moving Parkinson’s disease patient. *Mov. Disord.* **34**, 757–759 (2019). 30892717 10.1002/mds.27657 PMC6593659

[20] Kehnemouyi, Y. M. et al. Modulation of beta bursts in subthalamic sensorimotor circuits predicts improvement in bradykinesia. *Brain* **144**, 473–486 (2021). 33301569 10.1093/brain/awaa394 PMC8240742

[21] Darcy, N. et al. Spectral and spatial distribution of subthalamic beta peak activity in Parkinson’s disease patients. *Exp. Neurol.* **356**, 114150 (2022). 35732220 10.1016/j.expneurol.2022.114150

[22] An, Q. et al. Adaptive deep brain stimulation for Parkinson’s disease: looking back at the past decade on motor outcomes. *J. Neurol.* **270**, 1371–1387 (2022). 36471098 10.1007/s00415-022-11495-z

[23] Guidetti, M. et al. Clinical perspectives of adaptive deep brain stimulation. *Brain Stimul.* **14**, 1238–1247 (2021). 34371211 10.1016/j.brs.2021.07.063

[24] Little, S. & Brown, P. Debugging adaptive deep brain stimulation for Parkinson’s disease. *Mov. Disord.* **35**, 555–561 (2020). 32039501 10.1002/mds.27996 PMC7166127

[25] Teymourian, H. et al. Closing the loop for patients with Parkinson disease: where are we? *Nat. Rev. Neurol.* **18**, 497–507 (2022). 35681103 10.1038/s41582-022-00674-1

[26] Pina-Fuentes, D. et al. Acute effects of adaptive Deep Brain Stimulation in Parkinson’s disease. *Brain Stimul.* **13**, 1507–1516 (2020). 32738409 10.1016/j.brs.2020.07.016 PMC7116216

[27] Little, S. et al. Adaptive deep brain stimulation in advanced Parkinson disease. *Ann. Neurol.* **74**, 449–457 (2013). 23852650 10.1002/ana.23951 PMC3886292

[28] Rosa, M. et al. Adaptive deep brain stimulation in a freely moving Parkinsonian patient. *Mov. Disord.* **30**, 1003–1005 (2015). 25999288 10.1002/mds.26241 PMC5032989

[29] Bocci, T. et al. Eight-hours conventional versus adaptive deep brain stimulation of the subthalamic nucleus in Parkinson’s disease. *NPJ Parkinsons Dis.* **7**, 88 (2021). 34584095 10.1038/s41531-021-00229-z PMC8478873

[30] Velisar, A. et al. Dual threshold neural closed loop deep brain stimulation in Parkinson disease patients. *Brain Stimul.* **12**, 868–876 (2019). 30833216 10.1016/j.brs.2019.02.020

[31] Gilron, R. et al. Long-term wireless streaming of neural recordings for circuit discovery and adaptive stimulation in individuals with Parkinson’s disease. *Nat. Biotechnol.* **39**, 1078–1085 (2021). 33941932 10.1038/s41587-021-00897-5 PMC8434942

[32] Rosa, M. et al. Adaptive deep brain stimulation controls levodopa-induced side effects in Parkinsonian patients. *Mov. Disord.* **32**, 628–629 (2017). 28211585 10.1002/mds.26953 PMC5412843

[33] Petrucci, M. N. et al. Neural closed-loop deep brain stimulation for freezing of gait. *Brain Stimul.* **13**, 1320–1322 (2020). 32634599 10.1016/j.brs.2020.06.018 PMC8189032

[34] Arlotti, M. et al. Eight-hours adaptive deep brain stimulation in patients with Parkinson disease. *Neurology* **90**, e971–e976 (2018). 29444973 10.1212/WNL.0000000000005121 PMC5858949

[35] Little, S. et al. Adaptive deep brain stimulation for Parkinson’s disease demonstrates reduced speech side effects compared to conventional stimulation in the acute setting. *J. Neurol. Neurosurg. Psychiatry* **87**, 1388–1389 (2016). 27530809 10.1136/jnnp-2016-313518 PMC5136720

[36] Little, S. et al. Bilateral adaptive deep brain stimulation is effective in Parkinson’s disease. *J. Neurol. Neurosurg. Psychiatry* **87**, 717–721 (2016). 26424898 10.1136/jnnp-2015-310972 PMC4941128

[37] Nakajima, A. et al. Case Report: Chronic adaptive deep brain stimulation personalizing therapy based on Parkinsonian state. *Front Hum. Neurosci.* **15**, 702961 (2021). 34483867 10.3389/fnhum.2021.702961 PMC8414587

[38] Plate, A. et al. Peaks in the beta band of the human subthalamic nucleus: a case for low beta and high beta activity. *J. Neurosurg.* **136**, 672–680 (2022). 34560646 10.3171/2021.3.JNS204113

[39] Shreve, L. A. et al. Subthalamic oscillations and phase amplitude coupling are greater in the more affected hemisphere in Parkinson’s disease. *Clin. Neurophysiol.* **128**, 128–137 (2017). 27889627 10.1016/j.clinph.2016.10.095

[40] Tinkhauser, G. et al. Beta burst dynamics in Parkinson’s disease OFF and ON dopaminergic medication. *Brain* **140**, 2968–2981 (2017). 29053865 10.1093/brain/awx252 PMC5667742

[41] Anidi, C. et al. Neuromodulation targets pathological not physiological beta bursts during gait in Parkinson’s disease. *Neurobiol. Dis.* **120**, 107–117 (2018). 30196050 10.1016/j.nbd.2018.09.004 PMC6422345

[42] Sirica, D. et al. Neurophysiological biomarkers to optimize deep brain stimulation in movement disorders. *Neurodegener. Dis. Manag.* **11**, 315–328 (2021). 34261338 10.2217/nmt-2021-0002 PMC8977945

[43] Goyal, A. et al. The development of an implantable deep brain stimulation device with simultaneous chronic electrophysiological recording and stimulation in humans. *Biosens. Bioelectron.* **176**, 112888 (2021). 33395569 10.1016/j.bios.2020.112888 PMC7953342

[44] Koss, A. M., Alterman, R. L., Tagliati, M. & Shils, J. L. Calculating total electrical energy delivered by deep brain stimulation systems. *Ann. Neurol.* **58**, 168 (2005). 15984018 10.1002/ana.20525

[45] Stanslaski, S. et al. A chronically implantable neural coprocessor for investigating the treatment of neurological disorders. *IEEE Trans. Biomed. Circuits Syst.* **12**, 1230–1245 (2018). 30418885 10.1109/TBCAS.2018.2880148 PMC6415546

[46] Jimenez-Shahed, J. Device profile of the percept PC deep brain stimulation system for the treatment of Parkinson’s disease and related disorders. *Expert Rev. Med. Devices* **18**, 319–332 (2021). 33765395 10.1080/17434440.2021.1909471

[47] Technical Considerations for Medical Devices with Physiologic Closed-Loop Control Technology | Food and Drug Administration. Located at https://www.fda.gov/regulatory-information/search-fda-guidance-documents/technical-considerations-medical-devices-physiologic-closed-loop-control-technology (2023).

[48] Goetz, C. G. et al. Movement Disorder Society‐sponsored revision of the Unified Parkinson’s Disease Rating Scale (MDS‐UPDRS): scale presentation and clinimetric testing results. *Mov. Disord.* **23**, 2129–2170 (2008). 19025984 10.1002/mds.22340

