# Supplementary material for: Sensing data and methodology from the Adaptive DBS Algorithm for Personalized Therapy in Parkinson’s Disease (ADAPT-PD) clinical trial
Source: NPJ Parkinsons Dis. 2024 Sep 17;10:174. doi: 10.1038/s41531-024-00772-5 (PMC11408616; doi:10.1038/s41531-024-00772-5)
Supplement: Supplementary file 1 — Supplementary consortia list and supplemental table. [file 41531_2024_772_MOESM1_ESM.pdf]

List of consortia members.

Principal Investigator	Sub-Investigators	Institution
Helen Bronte Stewart, MD, MSE<br>*(Global PI)*	Gaurav Chattree, MD	Stanford University School of Medicine
Rob De Bie, MD, PhD	Martijn Beudel, MD, PhD<br>Martijn de Neeling, MD<br>Dan Pina Fuentes, MD<br>Peter Richard Schuurman, MD, PhD<br>Bart Swinnen, MD	Amsterdam UMC – locatie AMC
Alfonso Fasano, MD, PhD	Alex Boogers, MD, PhD<br>Qais Sadi, MD*	Toronto Western Hospital
Michal Gostkowski, DO	Umar Shuaib, MD	Cleveland Clinic
Travis Hassell, MD, PhD	Fenna Phibbs, MD, MPH<br>Englot Darrio, MD, PhD	Vanderbilt University Medical Center
Todd Herrington, MD, PhD	Mark Richardson, MD, PhD, FAANS<br>Alice Flaherty, MD, PhD<br>Skyla Lynch, BS	Massachusetts General Hospital
Kyle Mitchell, MD	Jeff Cooney, MD<br>Dennis Turner, MA, MD	Duke University Medical Center
Elena Moro, MD, PhD	Sara Meoni, MD<br>Anna Castrioto, MD<br>Valerie Fraix, MD	Centre Hospitalier Universitaire de Grenoble - Site Nord
Jill Ostrem, MD	Simon Little, MBBS, MRCP, PhD<br>Philip Starr, MD, PhD<br>Lauren Hammer, MD, PhD*<br>Hengameh Zahed, MD, PhD*	University of California, San Francisco Medical Center
Adolfo Ramirez-Zamora, MD<br><br>Leonardo Almeida, MD*	Kelly Foote, MD<br>Coralie de Hemptinne MS, PhD	University of Florida<br>Health Shands Hospital
** denotes previous principal investigator or sub-investigator that has transitioned to a new institution or left the trial. Investigators are listed by institutions where the clinical trial work was completed. Only enrolling sites listed here.*		

Supplemental Table 1. Inclusion and Exclusion Criteria

Inclusion Criteria	Exclusion Criteria
1. Subject has idiopathic Parkinson's disease	1. Subject and/or caregiver is unable to utilize the patient programmer
2. Subject is implanted with Percept PC (Model B35200) and Medtronic DBS leads (Model 3387, 3389, B33005 or B33015) and extensions (Model 37085, 37086 or B34000) bilaterally in the same target (physician confirmed), STN or GPi. Legacy leads = Model 3387, 3389. **Revised for directional cohort:** Subject is implanted with Percept PC (Model B35200) and Medtronic SenSight DBS leads (Model B33005 or B33015) and extensions (Model B34000) bilaterally in the same target (physician confirmed), STN or GPi	2. Subject has more than one lead in each hemisphere of the brain
3. In the opinion of the investigator, the subject responds to DBS Therapy	3. Subject has cortical leads or additional unapproved hardware implanted in the brain
4. Based on the opinion of the investigator, the subject's cDBS parameters and PD medications are stable and expected to remain stable from enrollment through the end of the aDBS Evaluation Phase	4. Subject has more than one INS
5. Subject is configured to ring mode monopolar or dual monopolar stimulation using contacts 1 and/or 2 (9 and/or 10) on at least one side. **Revised for directional cohort:** Subject is configured to directional monopolar or dual monopolar stimulation using contacts 1 and/or 2 (9 and/or 10)	5. At enrollment, the subject's INS has a predicted battery life of <1 year
6. Subject is willing and able to attend all study-required visits and complete the study procedures (e.g. 1-month recall questionnaires, MDS-UPDRS III)	6. Subject has Beck Depression Inventory II (BDI-II) > 25
7. Subject has the ability to understand and provide written informed consent for participation in the study prior to the study-related procedures being conducted	7. Subject requires diathermy, transcranial magnetic stimulation (TMS), or electroconvulsive therapy (ECT)
8. Subject is a male or non-pregnant female. If female of child-bearing potential, and if sexually active, must be using, or agree to use, a medically-acceptable method of birth control as confirmed by the investigator	8. Subject has a metallic implant in the head, (eg, aneurysm clip, cochlear implant)
9. For subjects with the SenSight system: Subject is configured to the following stimulation rates: 55, 85, 110, 125, 145, 164 or 180 Hz (as required for sensing/aDBS)	9. Subject has, or plans to obtain, an implanted electrical stimulation medical device anywhere in the body (eg, cardiac pacemaker, defibrillator, spinal cord stimulator)
LFP Screening Criteria (start of cDBS Baseline visit): Subject has peak frequency band (8-30 Hz, amplitude ≥ 1.2 μVp) detected on either left and/or right DBS leads on sensing channels 0-2, 0-3, or 1-3; 8-10, 8-11, or 9-11	10. Subject has, or plans to obtain, an implanted medication pump for the treatment of Parkinson's disease (eg, DUOPA™ infusion pump) and/or portable infusion pump
	11. Based on the opinion of the investigator, the subject has an abnormal neurological examination that would preclude them from study participation

12. Subject is breast feeding
13. Subject is under the age of 18 years
14. Subject is currently enrolled in or plans to enroll in any concurrent drug and/or device study that may confound the results of this study as determined by the Medtronic study team
15. Subject is unable to use or tolerate wearable
16. Subjects with signal artifact on all 6 aDBS sense pathways (3 each on both DBS leads) which preclude the clinician from setting thresholds
